# Supplementary material for: Vapour-phase-transport rearrangement technique for the synthesis of new zeolites
Source: Nat Commun. 2019 Nov 12;10:5129. doi: 10.1038/s41467-019-12882-3 (PMC6851152; doi:10.1038/s41467-019-12882-3)
Supplement: Supplementary file 1 — Supplementary Information [file 41467_2019_12882_MOESM1_ESM.pdf]

Supplementary Information

Vapour-phase-transport rearrangement technique for the synthesis of new zeolites

Kasneryk et al.

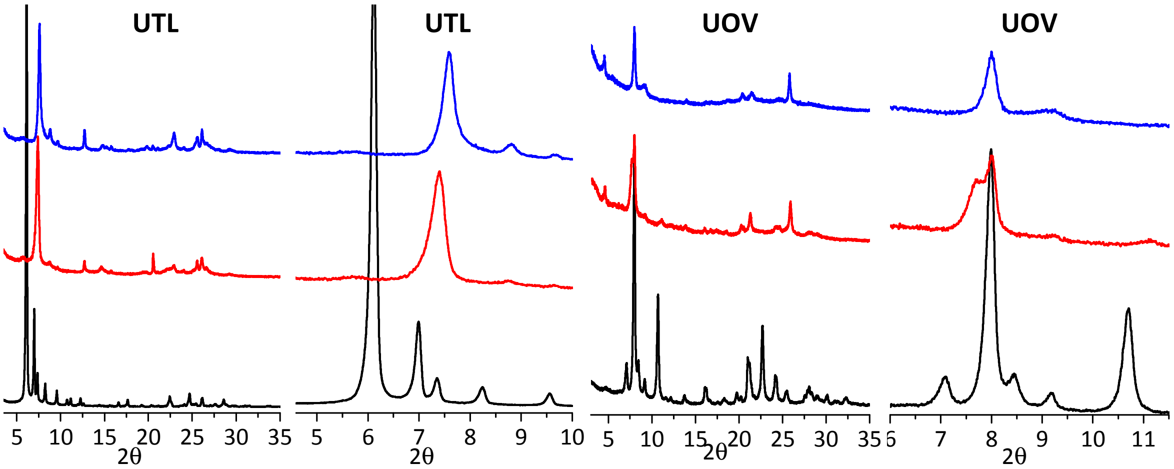

Supplementary Figure 1. XRD patterns of the UTL- and UOV-derived zeolites. The figure shows powder XRD patterns of **UTL** (Si/Ge = 2) and **UOV** (Si/Ge = 1.5) zeolites (black lines); samples subjected to VPT (red lines) and further calcination (blue lines). Two types of 2θ scales are used for clarity. Treatment of **UTL** leads to the formation of the IPC-7 material (change of first line position: red vs. blue patterns). Transformation of UOV results in the formation of the IPC-12 zeolite (black vs. red/blue patterns).

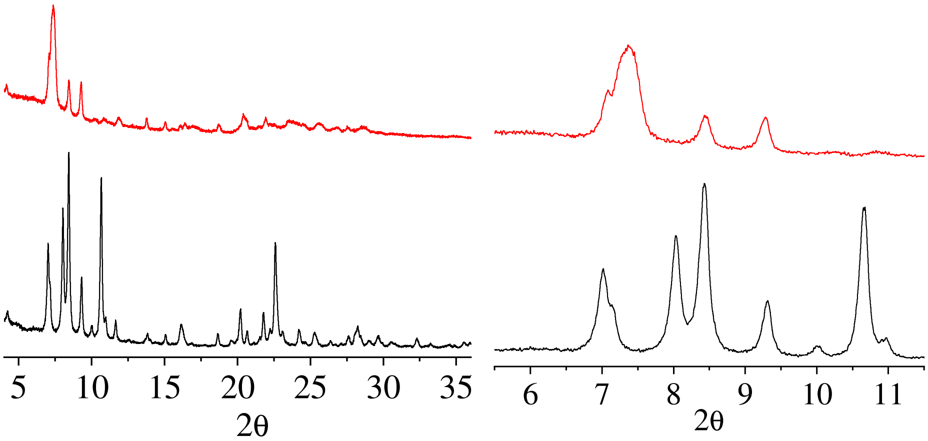

Supplementary Figure 2. XRD of initial IWW and respective VPT intermediate. XRD patterns of the initial **IWW** (black) and IPC-18P (red). Two types of 2θ scales are used for clarity.
Layers of IWW zeolite (high 2θ region in XRD pattern) being unstable during conventional ADOR treatment, withstand VPT resulting in intermediate derivative (IPC-18P).

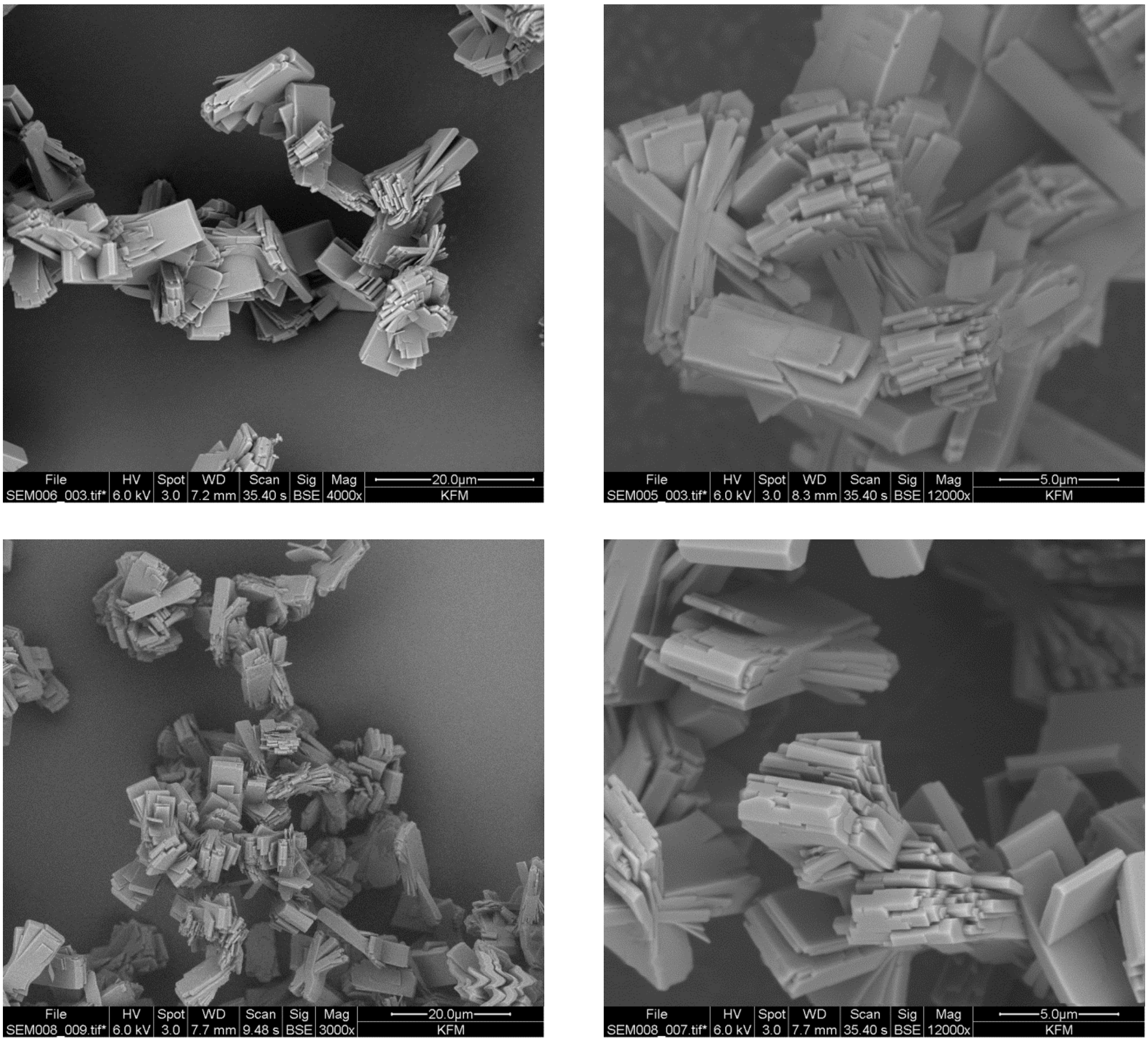

Supplementary Figure 3. Comparison of the morphology of initial IWW and final IPC-18. SEM images of the initial **IWW** (top) and IPC-18 (bottom). SEM images show similar shape and size of the crystals for both parent and daughter zeolites indicating no significant change of crystal morphology during VPT.

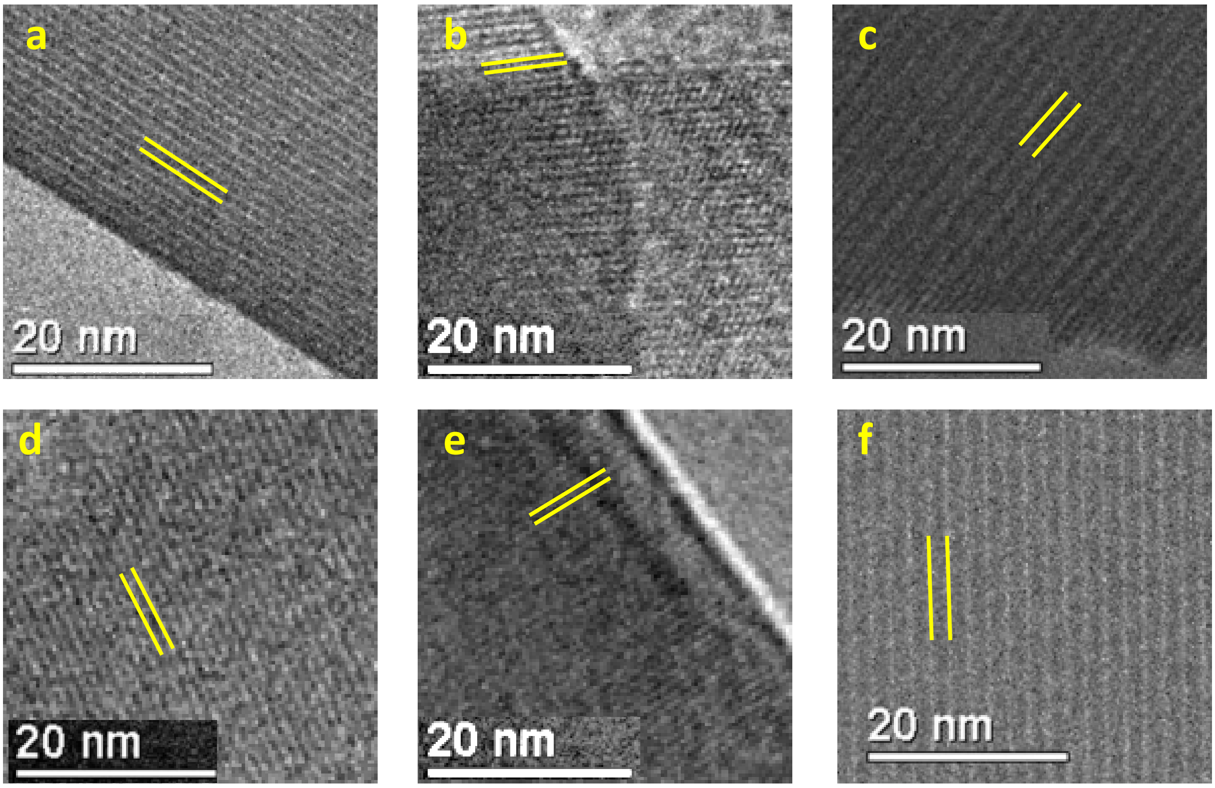

Supplementary Figure 4. Comparison of the TEM images of IWW and IPC-18. HRTEM images of initial **IWW** (a-c): d-spacings = 1.20 nm (001 projection, a), 0.95 nm (010, b), 2.02 nm (100, c); and final IPC-18 (d-f): d-spacing = 1.20 nm (001, d), d-spacing = 0.90 nm (010, e), d-spacing = 1.92 nm (100, f). Images collected in three projections confirm the major decrease of d-spacing along 100 projection and minor change along 010 direction, while d-spacing along 001 remains almost the same. This confirms anisotropic structural transformation of the structure of IWW during VPT.

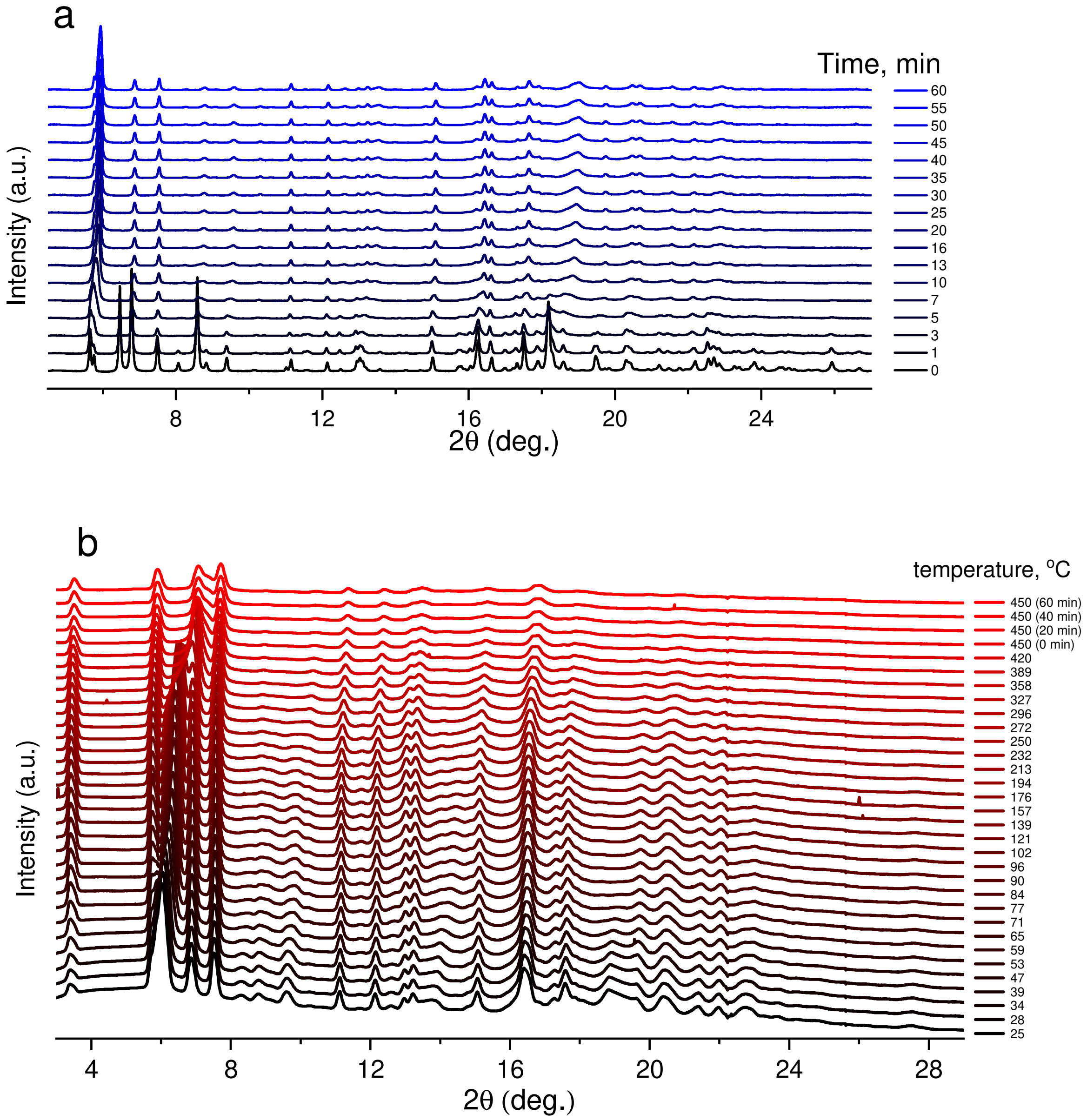

Supplementary Figure 5. In situ XRD patterns for VPT (full 2θ range). Full *in situ* XRD profiles for hydrolysis (a) and calcination (b). Selected areas of the patterns (Fig. 4) are analyzed and discussed in the main part.

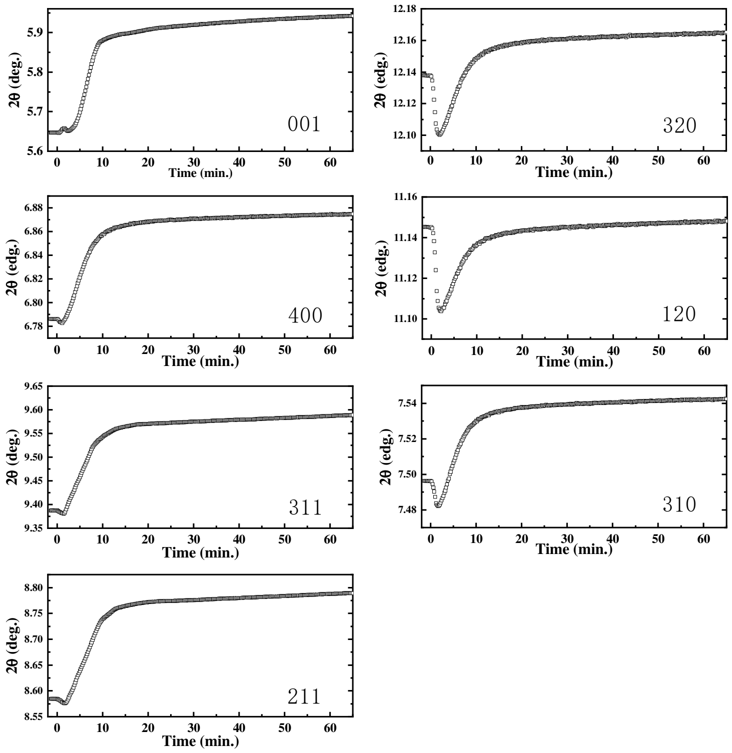

Supplementary Figure 6. Time-dependent peak position of (001), (320), (400), (120), (311), (310) and (211) reflections during the hydrolysis process. Selected parameters are plotted (Fig. 4) and discussed in the main part.

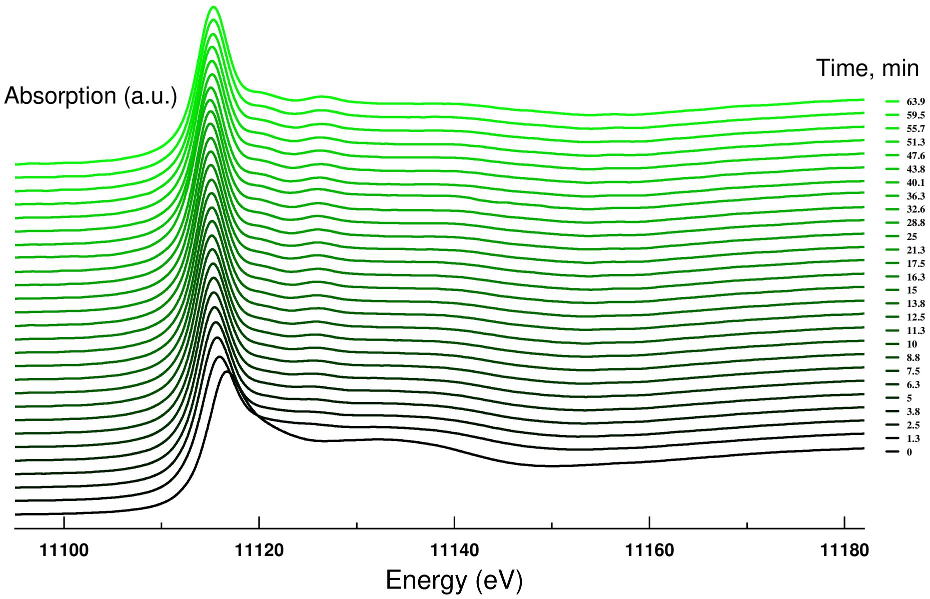

Supplementary Figure 7. Full *in situ* Ge XANES profiles. Patterns were used for quantitative analysis discussed in the main part (Fig. 4).
